# Phase Transitions in Two-Dimensional Systems of Janus-like Particles on a Triangular Lattice

**DOI:** 10.3390/ijms221910484

**Published:** 2021-09-28

**Authors:** Andrzej Patrykiejew

**Affiliations:** Department of Theoretical Chemistry, Institute of Chemical Sciences, Faculty of Chemistry, MCS University, 20031 Lublin, Poland; andrzej.patrykiejew@poczta.umcs.lublin.pl

**Keywords:** Janus particles, phase transitions, Monte Carlo simulation

## Abstract

We studied the phase behavior of two-dimensional systems of Janus-like particles on a triangular lattice using Monte Carlo methods. The model assumes that each particle can take on one of the six orientations with respect to the lattice, and the interactions between neighboring particles were weighted depending on the degree to which their A and B halves overlap. In this work, we assumed that the AA interaction was fixed and attractive, while the AB and BB interactions varied. We demonstrated that the phase behavior of the systems considered strongly depended on the magnitude of the interaction energies between the AB and BB halves. Here, we considered systems with non-repulsive interactions only and determined phase diagrams for several systems. We demonstrated that the phase diagram topology depends on the temperature at which the close-packed systems undergo the orientational order–disorder transition.

## 1. Introduction

Janus particles have a surface composed of two chemically different patches, A and B [1,2]. The surface chemical anisotropy, which can be tuned by an appropriate fictionalization, results in orientation-dependent interactions. The chemical composition and the size of patches, influence both self-assembly and the formation of different ordered structures in two- and three-dimensional systems [3,4,5,6,7,8]. In the region of low and moderate densities, the formation of micelles, vesicles, and worm-like clusters has been observed [9,10,11]. It was also shown that Janus particles form crystals of different structures and density [11].

The behavior of dense two-dimensional systems of Janus particles has been recently studied by several authors [3,12,13,14]. Shin and Schweizer [3] used the Kern–Frenkel model [15] and developed a version of self-consistent phonon theory, which predicted the formation of different orientationally ordered hexagonal phases. The structure of these phases was found to be primarily determined by the so-called Janus balance [16], defined by the size of the attractive patch. Shin and Schweizer showed also that such systems may undergo phase transitions between different orientationally ordered phases. Similar orientationally ordered structures were observed by Iwashita and Kimura [4]. On the other hand, experimental study and Monte Carlo simulation of Jiang et al. [5] showed the formation of a glass-like phase, instead of the theoretically predicted zigzag phase [3].

In our recent paper [13], we studied the orientational order–disorder transitions in closely packed two-dimensional systems of Janus particles, using a simple lattice model, which allowed for only six different orientations of each particle. It was demonstrated that the nature of the transition is entirely determined by the sign of the parameter $\epsilon =u_{AA}+u_{BB}-2u_{AB}$, where $u_{AA}$, $u_{AB}$ and $u_{BB}$ are the energies of interaction between the nearest neighbor pairs with their AA, AB, and BB halves facing one another. The parameter ϵ determines whether the contacts between the like (AA and BB) or unlike (AB) halves are favored.

When ϵ<0, the systems were shown to order into the zigzag phase, with the order–disorder transition belonging to the universality class of the three-state Potts model [17]. On the other hand, when ϵ>0, the ordered phase was found to be different, with the order–disorder transition belonging to the universality class of the four-state Potts model [17]. Here, we should mention that various lattice models (Ising, Potts, etc.) are commonly used to describe the behavior of diverse physical systems [18,19].

We also studied the phase behavior of Janus-like particles [14] using the same lattice model with $u_{AB}=u_{BB}=0$, i.e., with the interaction potential similar to that proposed by Kern and Frenkel [15]. The model was studied using the Monte Carlo method in the grand canonical ensemble, and two versions of the model were considered. In the first version, the strength of attractive interaction, confined to the A halves of neighboring particles, was assumed to depend on the degree to which they were overlapping. In the second version, it was assumed that the interaction energy between a pair of neighboring particles was the same for any mutual orientations, in which their A patches overlapped to any extent.

It was demonstrated that both versions of the model led to qualitatively different results. In the case of the first version, the self-assembly was found to lead to different stripped structures, depending on the density and the temperature. In particular, we found that, at sufficiently low temperatures, the condensation led from a very dilute lamellar gas phase to the high density ordered zigzag phase. At intermediate temperatures, the system underwent two first-order phase transitions. The first led to the condensation of the gas phase into the partially ordered, phase ($Z_{2}$), with kinked stripes that were predominantly ordered along two axes of the lattice.

The second transition occurred between the $Z_{2}$ phase and the high density well-ordered zigzag phase (*Z*). At sufficiently high temperatures, only one continuous transition, between the disordered fluid-like and the ordered zigzag phases, was observed. In the case of the second model, we found only one first-order transition at low temperatures. This transition occurs between a dilute gas-like phase and the ordered phase, which forms a kagome lattice of the density equal to 6/7. A further increase of the density was demonstrated to lead to the reorientation of particles and the formation of dense glass-like structure, similar to that observed by Jiang et al. [5].

Thus, the phase behavior was demonstrated to be sensitive to the magnitude of attractive interaction acting between differently oriented particles.

The primary aim of this work is to discuss the phase behavior of two-dimensional systems of Janus-like particles with the tuned interactions between their different parts. In particular, we were interested in the question of how the phase behavior is affected by the stability of the dense ordered phase and by the strength of attractive interactions between particles.

To this end, we applied the same lattice gas model as used in [13,14,20] and assumed that the interaction between the neighboring Janus particles depends on the degrees to which their different parts overlap. We considered three series of systems in which the A-A interaction was fixed while A-B and B-B interactions were varied. In the first series, the AB and BB interactions were assumed to be the same, $u_{AB}=u_{BB}=u^{*}$. In the second (third) series, $u_{AB}$ ($u_{BB}$) was assumed to be equal to zero, while $u_{BB}$ ($u_{AB}$) was varied.

Only the second series, with $u_{AB}=0$, appeared to mimic real Janus particles with hydrophilic and hydrophobic parts [2,3,14]. However, the other two series are also of interest, since each of them has demonstrated a little different phase behavior.

## 2. The Model and Methods

**Figure 1:**
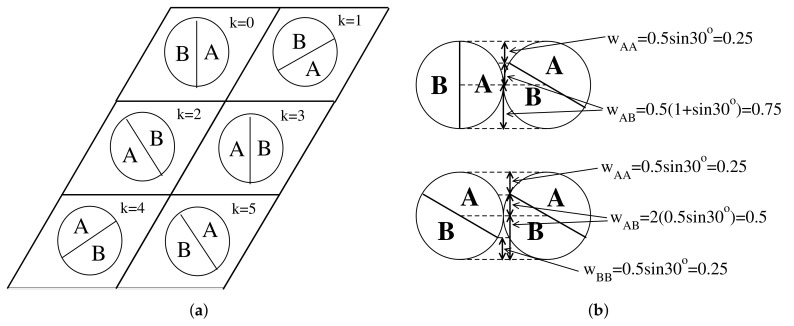
(**a**) The six possible orientations of a particle on a triangular lattice. (**b**) Two representative examples of differently oriented Janus particles, with (k,l)=(1,6) and (6,6) and the separation vector $r=a_{1}$, demonstrating how the weights determining the interaction energy between neighboring particles were calculated.

As already mentioned, the model used here is quite similar that considered in [13,14,20]. Thus, the Janus particles placed on a triangular lattice were assumed to be made of two halves A and B, and each particle was assumed to take on one of the six orientations, defined by the angle θ(k)=(k−1)(2π/6) (k=1,…,6), measured with respect to the *x*-axis (see Figure 1a). Throughout this work, we assumed that all interactions were short-ranged and limited to the first nearest neighbors.

The interaction energy between a pair of particles located on adjacent sites *i* and *j*, $u(k_{i},k_{j},r_{ij})$, was assumed to depend on their orientations, $k_{i}$ and $k_{j}$, defined by the angles, $\theta (k_{i})$ and $\theta (k_{j})$, as well as on the separation vector, $r_{ij}$, of unit length. The triangular lattice is described by three unit vectors: $a_{1}=\left(1,0\right)$, $a_{2}=\left(0.5,\sqrt{3}/2\right)$, and $a_{3}=\left(-0.5,\sqrt{3}/2\right)$, and hence there are six different separation vectors, which are equal to $\pm a_{i}$ (i=1,2,3).

To each particle, we assigned the spin vector of unit length, S=(cos(θ),sin(θ)), and hence $u(k_{i},k_{j},{\vec{r}}_{ij})$ can be written as $u(S_{i},S_{j},r_{ij})$. Moreover, we assumed that the energy of interaction between a pair of neighboring particles depends on the degree to which their various halves overlap. This leads to the following expression for $u(S_{i},S_{j},r_{ij})$:

(1) $$u\left(S_{i},S_{j},r_{ij}\right)=w_{AA}\left(S_{i},S_{j},r_{ij}\right)u_{AA}+w_{AB}\left(S_{i},S_{j},r_{ij}\right)u_{AB}+w_{BB}\left(S_{i},S_{j},r_{ij}\right)u_{BB}\;\;,$$

where $u_{AA}$, $u_{AB}$, and $u_{BB}$ are the interaction energies corresponding to the orientations, in which the AA, AB or BB halves face one another, while $w_{AA}\left(S_{i},S_{j},r_{ij}\right)$, $w_{AB}\left(S_{i},S_{j},r_{ij}\right)$, and $w_{BB}\left(S_{i},S_{j},r_{ij}\right)$ are the weights, determined by the degrees to which the AA, AB, and BB regions overlap for given relative orientations, specified by $S_{i}$ and $S_{j}$, and locations, specified by the separation vector $r_{ij}$ (see Figure 1b). There are 12 different values of the pair interaction energy, as summarized in Table 1. In Table 1, we also give the orientations of pairs of neighboring particles corresponding to different values of $u(S_{i},S_{j},r_{ij})$, for $r_{ij}=a_{1}$.

To study the phase behavior, we used the Monte Carlo method in the grand canonical ensemble [21]. The Hamiltonian of the model reads

(2) $$H=\frac{1}{2}\sum_{i,j}u\left(S_{i},S_{j},r_{ij}\right)n_{i}n_{j}-N\mu \;\;,$$

where the sum runs over all pairs of nearest neighbors; $n_{i}=1$, when the *i*-the site is occupied, and 0 otherwise; and *N* is the total number of particles in the system,

(3) $$N=\sum_{i=1}^{L^{2}}n_{i}$$

and μ is the chemical potential. In the system with linear dimension *L*, the total density is equal to $\rho =N/L^{2}$, and the densities of differently oriented particles are defined as

(4) $$\rho_{k}=\frac{1}{L^{2}}\sum_{i=1}^{L^{2}}n_{i}\delta \left(\theta \left(k_{i}\right)-\theta \left(k\right)\right).$$

Of course,

(5) $$\rho =\frac{1}{L^{2}}\sum_{l=1}^{6}\rho_{l}$$

Throughout this work, we assumed that $u_{AA}=-1.0$, with $|u_{AA}|$ taken as the unit of energy, while the values of $u_{AB}$ and $u_{BB}$ were varied. The temperature, the chemical potential, and all other energy-like quantities are expressed in the reduced units.

The simulations were carried out for rhomboid cells of the size L×L, with the standard periodic boundary conditions. Since the systems considered were found to form various ordered structures of different symmetry and density, we considered simulation cells of the sizes suitable to properly accommodate those structures in periodically repeated simulation cells.

The quantities recorded included the averages of the total density, 〈ρ〉, the densities of differently oriented particles, $\langle \rho_{k}\rangle$, the potential energy per site, 〈u〉, the heat capacity

(6) $$C_{V}=\frac{1}{T^{2}}\left[\langle H^{2}\rangle -{\langle H\rangle }^{2}\right],$$

and the density susceptibility per site

(7) $$\chi_{\rho }=\frac{1}{T}\left[\langle \rho^{2}\rangle -{\langle \rho \rangle }^{2}\right]\;\;.$$

To equilibrate the system, we used $10^{6}$–$10^{7}$ Monte Carlo steps and another $5\cdot 10^{6}$–$10^{8}$ Monte Carlo steps were used to calculate averages. Each Monte Carlo step involved $10\cdot L^{2}$ attempts to change the state of the system. In the grand canonical ensemble, the possible changes of the system state involved either the creation of a particle on a randomly chosen site, with also a randomly chosen orientation or the removal of a randomly chosen particle. The simulation at a given temperature usually began at a sufficiently low value of the chemical potential, corresponding to a very low density, and then the chemical potential was gradually increased up to the values at which the nearly entire lattice was filled.

After the recording of such an “ascending” isotherm, we performed the run, starting at a high density, and recorded the “descending“ isotherm. This procedure allowed locating the first-order phase transitions. In finite systems, the first-order transitions at low temperatures are usually accompanied by hysteresis loops, due to the presence of metastable states [21,22].

During the equilibration runs, the changes of the recorded quantities were monitored, and the equilibration was assumed to be complete when these quantities ceased to undergo systematic changes and showed only oscillations around average values. In some cases, this was achieved already after $10^{5}$–$5\cdot 10^{6}$ Monte Carlo steps; however, usually the equilibration required a considerably larger number of Monte Carlo steps, up to $10^{7}$.

## 3. Results and Discussion

**Figure 2:**
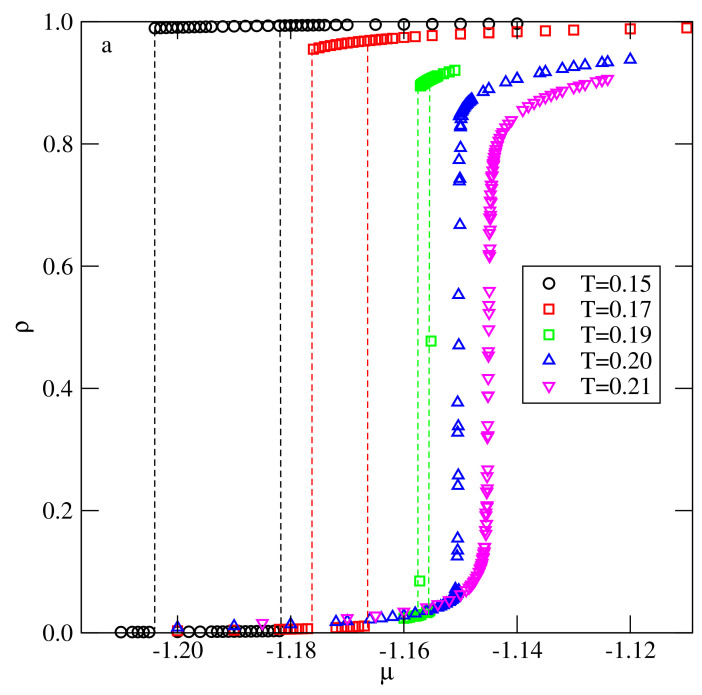
The examples of isotherms recorded for the system with $u^{*}=-0.1$ at different temperatures (given in the figure).

**Figure 3:**
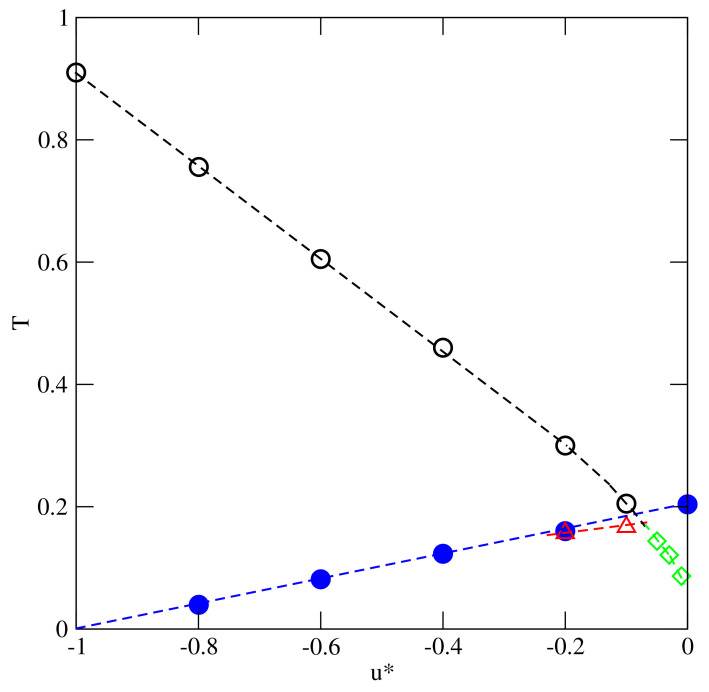
The changes of the critical temperature, $T_{c}\left(u^{*}\right)$, (circles), the critical end point temperature, $T_{cep}\left(u^{*}\right)$, (triangles) the orientational order−disorder transition temperature in close-packed systems, $T_{o}\left(u^{*}\right)$ (filled circles), and the tricritical point temperatures, $T_{trc}\left(u^{*}\right)$ (triangles), with $u^{*}$.

To begin, we consider the systems with $u_{AB}=u_{BB}=u^{*}$, assuming that $u^{*}\in \left[-1.0,-0.1\right]$. The isotherms, calculated at different temperatures and for different values of $u^{*}$, demonstrated that all these systems exhibit qualitatively the same behavior. Figure 2 presents the isotherms recorded for $u^{*}=-0.1,$ and the systems with $u^{*}<-0.1$ led to quite similar results and the presence of the first-order transition at sufficiently low temperatures. The transition can be treated as the gas–liquid condensation, and it terminates in the critical point. The critical temperature, $T_{c}\left(u^{*}\right)$, gradually increases when $u^{*}$ decreases from −0.1 to −1.0 (see Figure 3). In the particular case of $u^{*}=-1.0$, the critical temperature takes on the value of about 0.91, as predicted for the isotropic lattice gas model on a triangular lattice [23,24].

In [13,20], we show that the close-packed systems, with ρ=1.0 and different values of $u^{*}$, undergo the order–disorder transition between the orientationally disordered phase and the ordered zigzag (Z) phase. The transition was demonstrated to be continuous and belonging to the universality class of the three-state Potts model, with the transition temperature decreasing linearly to zero, when $u^{*}$ decreases toward −1.0. When $u^{*}=-1.0$, the interactions become isotropic, and hence no orientational order–disorder transition is possible. The locations of the orientational order–disorder transition, $T_{o}\left(u^{*}\right)$, are also included in Figure 3 and in all systems with $u^{*}\leq -0.1$ the critical temperature is higher than $T_{o}\left(u^{*}\right)$.

Therefore, at sufficiently low temperatures, the gas should condense directly into the ordered Z phase, while at higher temperatures, but still lower than $T_{c}\left(u^{*}\right)$, the condensation should lead to the orientationally disordered condensed phase. The transition between the dense orientationally disordered and the ordered zigzag phase is expected to be continuous, just the same as in the close-packed systems. This implies that the line of the orientational order–disorder transition terminates in the critical end point located on the condensed phase branch of the gas-condensed phase coexistence.

**Figure 4:**
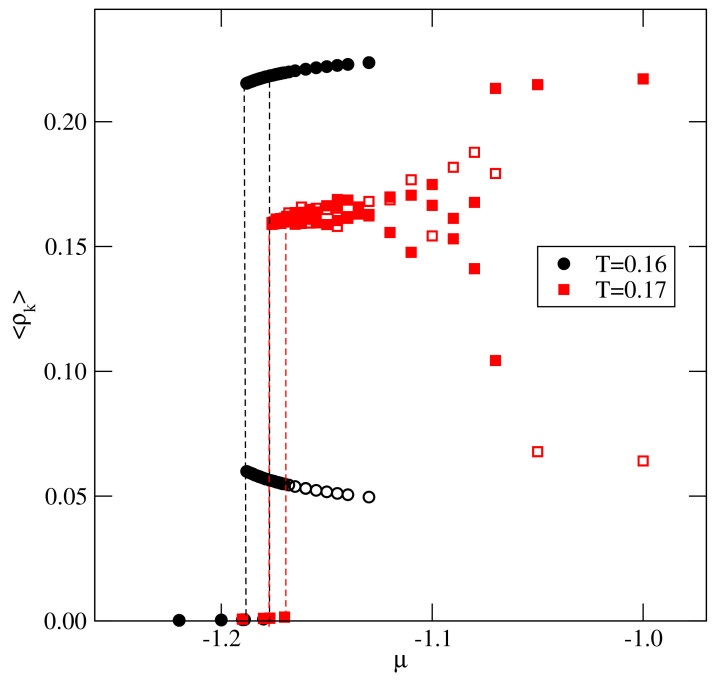
The densities of differently oriented particles along the isotherms obtained for the system with $u^{*}=-0.1$ at two temperatures, given in the figure. The filled (open) symbols correspond to the four favored (the two disfavored) orientations in the ordered zigzag structure.

This scenario was found in the systems characterized by $u^{*}=-0.1$ and −0.2, in particular, when $u^{*}=-0.1$. The recorded densities of differently oriented particles, $\langle \rho_{k}\rangle$, along the isotherms at T=0.16 and 0.17 demonstrated (see Figure 4) that, at T=0.16, the gas condensation led to the orientationally ordered Z phase, in which four orientations were favored (cf. [20]), while, at T=0.17, the gas condensation led to an orientationally disordered liquid.

**Figure 5:**
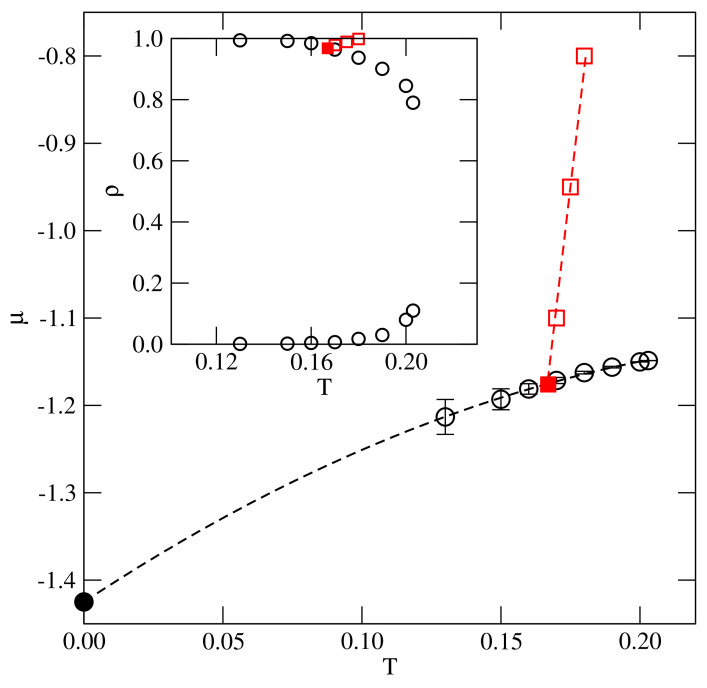
The estimated phase diagram for the system with $u^{|ast}=-0.1$. The main part shows the T−μ projection, and the inset gives the T−ρ projection. Circles and squares represent the coexistence points of the first−order and continuous transitions, respectively. The filled square marks the location of the critical end point, while the filled circle (in the main part) shows the location of the first-order transition in the ground state.

However, the orientationally disordered liquid phase undergoes the transition to the ordered Z phase at the chemical potential μ≈−1.12, and the density is equal to about 0.99. From the results obtained at different temperatures, we estimated the phase diagram for this system, which is given in Figure 5. As expected, the line of continuous orientational order–disorder transition meet the gas-condensed phase coexistence at the critical end point, located at $T_{cep}\left(-0.1\right)\approx 0.167$, $\mu_{cep}\left(-0.1\right)\approx -1.175$, and $\rho_{cep}\left(-0.1\right)\approx 0.965$. In the system with $u^{*}=-0.2$, the critical end point is located at $T_{cep}\left(-0.2\right)\approx 0.16$, $\mu_{cep}\left(-0.2\right)\approx -1.33$, and at the density $\rho_{cep}\left(-0.2\right)\approx 0.997$.

Qualitatively, the same behavior is bound to occur in the systems with lower values of $u^{*}$. However, since $T_{o}\left(u^{*}\right)$ decreases when $u^{*}$ becomes lower, the critical end point is shifted toward gradually decreasing temperatures, and toward the densities very close to unity. Already in the system with $u^{*}=-0.2$, the estimated density at the critical end point is very high.

**Figure 6:**
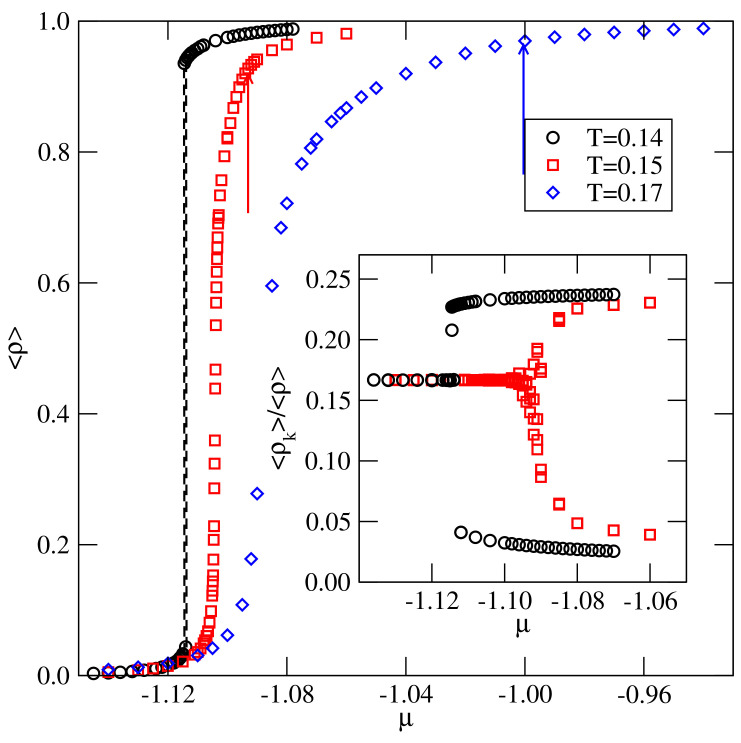
The main part shows the isotherms recorded at different temperatures for the system with $u^{*}=-0.05$, while the inset presents the changes of the ratio $\langle \rho_{k}\rangle /\langle \rho \rangle$ along the isotherms at T=0.14 and 0.15. The arrows in the main part mark the locations of the orientational order−disorder transition.

The situation changes when $u^{*}$ becomes higher than about −0.084, since the order–disorder transition temperatures, $T_{o}\left(u^{*}\right)$, exceed the expected critical temperatures (cf. Figure 3). This does not exclude the possibility that the phase diagrams may still look like those shown in Figure 5, however. The calculations carried out for the systems with $u^{*}=-0.05$ and −0.03 demonstrated a different behavior. The isotherms (see the main part of Figure 6) and the isothermal changes of the ratio $\langle \rho_{k}\rangle /\langle \rho \rangle$ (see the inset to Figure 6) obtained for the system with $u^{*}=-0.05$, demonstrated that, at the temperature T=0.14, the first-order transition leads from the gas phase directly to the well developed Z structure.

At higher temperatures of T=0.15 and 0.17, which are still lower than $T_{o}\left(-0.05\right)\approx 0.19$, the isotherms do not show the first-order transition between the gas and liquid phases, and the density smoothly increases with μ. However, at sufficiently high densities, a continuous transition, associated with the development of orientationally ordered Z phase, takes place at temperatures up to $T_{o}\left(-0.05\right)$. At the temperatures above $T_{o}\left(-0.05\right)$, we did not observe the formation of the Z phase at all.

The orientational order–disorder transition is not accompanied by any visible density anomalies along the isotherms, but it leads to large changes of the ratio $\langle \rho_{k}\rangle /\langle \rho \rangle$ (cf. the inset to Figure 6). In the orientationally disordered phase, all six orientations are equally probable, and hence $\langle \rho_{k}\rangle /\langle \rho \rangle \approx 1/6$. In the orientationally ordered phase, four orientations are favored, while the remaining two are disfavored. The transition is also accompanied by the appearance of heat capacity peaks of the height and location of maxima depending on the simulation cell size.

**Figure 7:**
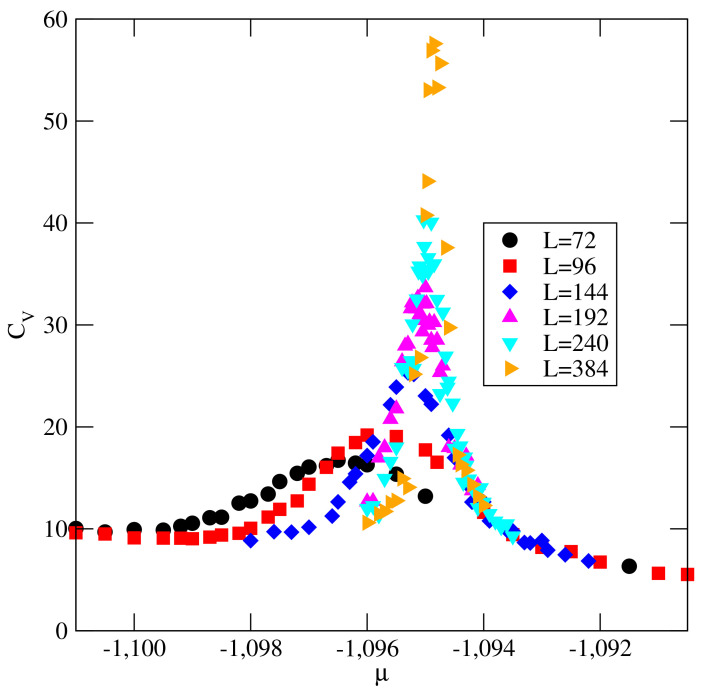
The heat capacity curves for the system with $u^{*}=-0.05$ at T=0.15, recorded using the simulation cells of different sizes (shown in the figure).

In Figure 7, we present examples of heat capacity curves obtained at T=0.15 and for different sizes of the simulation cell. Here, we should recall that, in the close-packed system, the orientational order–disorder transition belongs to the universality class of the three-state Potts model [12,20]. Therefore, the observed transition also belongs to the same universality class. However, to confirm this prediction, one would need to evaluate the size dependence of the joint distribution of density and energy fluctuations, since the scaling fields comprise mixtures of temperature and chemical potential [25,26].

**Figure 8:**
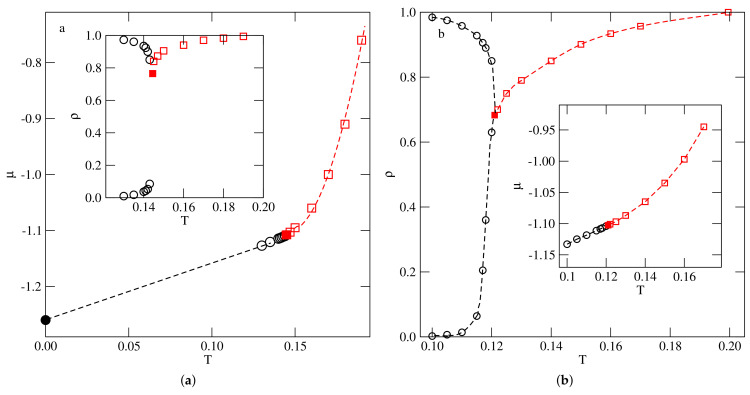
The phase diagrams evaluated for the systems with $u^{*}=-0.05$ (**a**), and −0.03 (**b**). Open circles and squares show the phase boundaries for the first−order and second−order transitions, respectively. The filled squares mark the locations of the tricritical points. In the main panel, the location of the gas–zigzag transition in the ground state is marked by the filled circle.

The constructed phase diagrams for the systems with $u^{*}=-0.05$ and −0.03 are shown in parts a and b of Figure 8, respectively. Taking into account that, in both systems, the coexistence lines of the first-order transition smoothly meet the lines of the continuous transition, we conclude that the first-order transition terminates in the tricritical point, $T_{trc}\left(u^{*}\right)$, which replaces the critical point. The estimated tricritical point temperatures in these two systems are $T_{trc}\left(-0.05\right)\approx 0.144$ and $T_{trc}\left(-0.03\right)\approx 0.12$.

**Figure 9:**
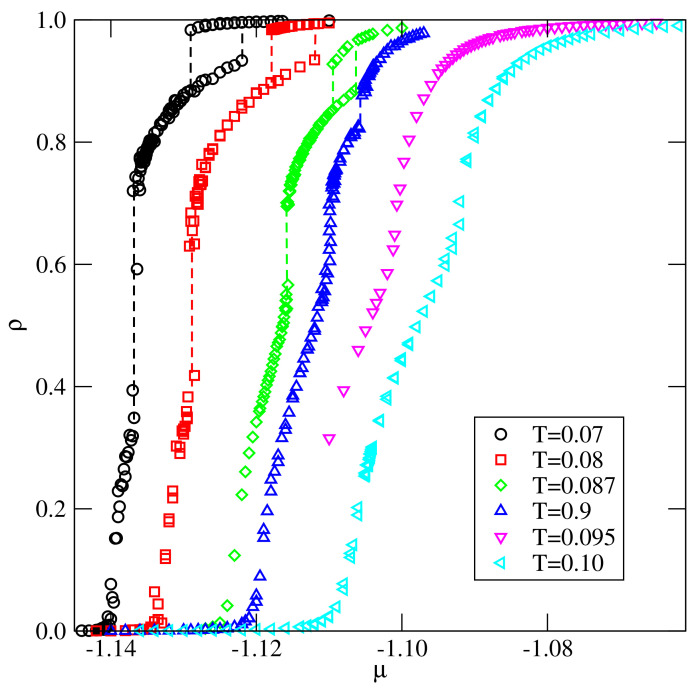
The isotherms obtained for the system with $u^{*}=-0.01$ at different temperatures.

**Figure 10:**
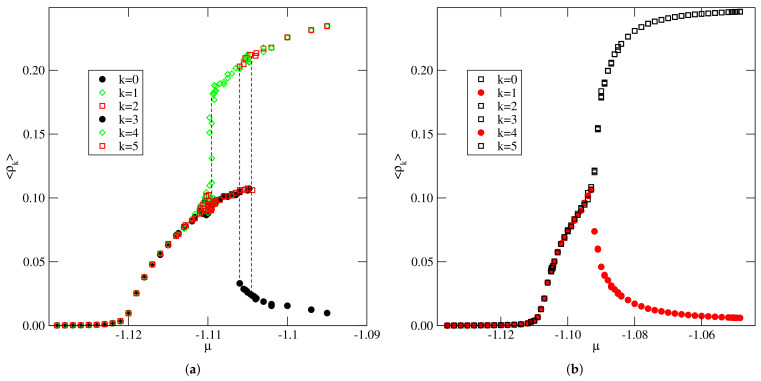
The changes of densities of differently oriented particles along the isotherm at T=0.09 (**a**) and T=0.10 (**b**), obtained for the system with $u^{*}=-0.01$. The dashed vertical lines in part (**a**) mark the locations of density jumps due to the transitions. One should note the hysteresis loop accompanying the transition between $Z_{2}$ and *Z* phases.

The calculations carried out for the system with $u^{*}=-0.01$, i.e., quite close to zero, demonstrated the behavior quite similar to that found in the case of $u^{*}=0.0$ [14]. Figure 9 shows a series of isotherms recorded for this system, which exhibit two discontinuous density jumps at temperatures between 0.07 and 0.09, indicating the presence of two first-order transitions. The first transition occurs between the orientationally disordered low density phase and the partially ordered phase, $Z_{2}$, in which two orientations are favored. This is illustrated by the changes of $\langle \rho_{k}\rangle$ along the isotherm at T=0.09 (see Figure 10a).

**Figure 11:**
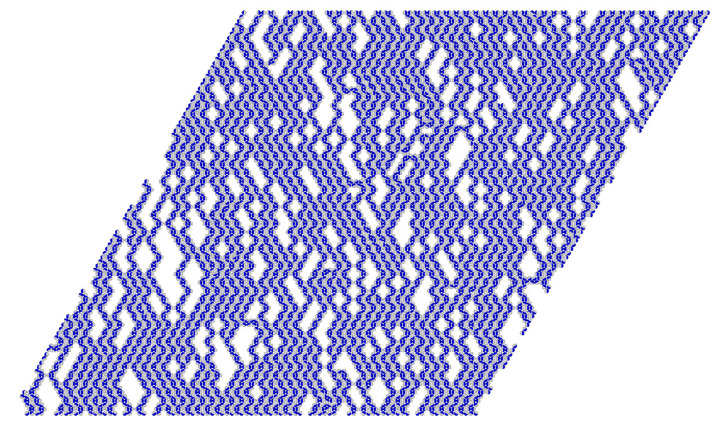
The snapshot recorded for the system with $u^{*}=-0.01$, at T=0.08 and μ=−1.126, which shows the structure of the phase $Z_{2}$.

The phase $Z_{2}$ consists of long kinked zigzag clusters, with a large number of 120^o^ kinks, predominantly oriented along with two out of three axes of the triangular lattice (see Figure 11). The second transition, which takes place at higher densities, leads to the development of the ordered Z phase. At temperatures T>0.09, only one continuous transition takes place. Figure 10b shows the changes of $\langle \rho_{k}\rangle$ along the isotherm at T=0.10 and demonstrates that the only transition occurs between the orientationally disordered (lamellar) fluid and the orientationally ordered Z phase.

**Figure 12:**
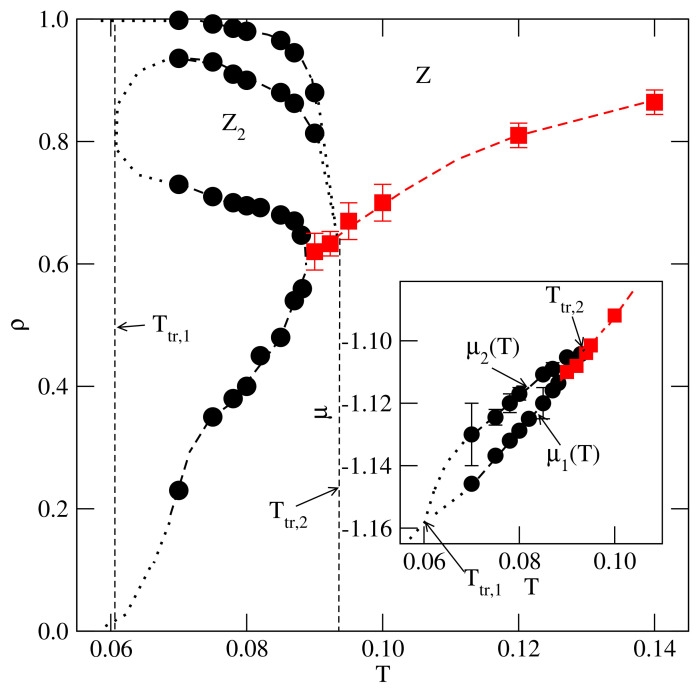
The proposed phase diagram for the system with $u^{*}=-0.01$. The main part and the inset show the T−ρ and the T−μ projections, respectively. The circles mark the first−order transitions, while the squares mark the locations of continuous transitions. The dotted vertical lines in the main panel mark the expected locations of the triple points.

The recorded isotherms, heat capacities, and density susceptibilities, allowed us to construct T−μ and T−ρ projections of the phase diagram as shown in Figure 12. Taking into account that, in the ground state, this system exhibits only one transition, between a gas-like and the ordered Z phases, we conclude that the triple point, $T_{tr,1}$, in which a very dilute gas-like phase coexists with the $Z_{2}$ and Z phases, must exist at a certain temperature below 0.07.

On the other hand, the lines of low and high density transitions, $\mu_{1}\left(T\right)$ and $\mu_{2}\left(T\right)$, are expected to meet at a temperature of about 0.093 and a density of about 0.65. At this point, the LF, $Z_{2}$, and Z phases coexist.

The change of the phase behavior between the systems with $u^{*}=-0.03$ and −0.01 results from the weakening of attractive AB and BB interactions. In the *Z* and $Z_{2}$ phases, every particle enjoys attractive interactions with all neighboring particles; however, the AB and BB attraction is weaker when $u^{*}=-0.01$. In the *Z* phase, the A half of each particle has contact with the A parts of four neighboring particles, and the formation of straight zigzag stripes is enhanced by the AB and BB attractions.

In the $Z_{2}$ phase, the A half of each particle interacts with either three or four A halves of neighboring particles, while the AB and BB attractive interactions are of lesser importance. On the other hand, the *Z* phase has a negligible residual entropy per particle in the thermodynamic limit [3], and this is stabilized by strong attraction. In the $Z_{2}$ phase, with large regions of empty sites, the entropy is higher than in the well-ordered Z phase. Thus, the phase $Z_{2}$ is stabilized by entropic effects.

However, the contribution of entropy to the free energy decreases when the temperature is lowered, and hence the structure of the condensed phase becomes dominated by the potential energy. This explains the appearance of the triple point $T_{tr,1}$. As the temperature increases, the ordering in both the *Z* and $Z_{2}$ phases is gradually destroyed by thermal fluctuations; however, the effect of these fluctuations is considerably stronger in the case of the already not well-ordered $Z_{2}$ phase. Therefore, the disordering of the $Z_{2}$ phase takes place at lower temperatures than the disordering of the *Z* structure, thus, leading to the presence of another triple point at the temperature $T_{tr,2}\approx 0.93$.

Now, we turn to the results obtained for a series of systems with $u_{AB}=0$, which are the most closely related to the usual models of Janus particles [15]. The close-packed systems with $u_{AB}=0$ and $u_{BB}<0$ undergo a continuous orientational transition, however, at temperatures increasing linearly from about 0.204, when $u_{BB}=0$, up to about 0.408, when $u_{BB}=-1.0$. Similarly, to the series with $u_{AB}=u_{BB}$, the phase behavior is expected to depend on the stability of the ordered Z phase.

The systems with $u_{BB}$ very close to zero were found to exhibit qualitatively the same behavior as the already discussed system with $u^{*}-0.01$ and the system with $u^{*}=0.0$. For lower $u_{BB}$, equal to −0.05 and −0.1, the phase diagrams were qualitatively the same as found for the systems with $u^{*}$ equal to −0.03 and −0.05. Thus, the dilute gas-like phase condenses directly into the zigzag ordered structure at the temperatures up to the tricritical point temperature, $T_{trc}\left(-0.05\right)\approx 0.135$ and $T_{trc}\left(-0.1\right)\approx 0.175$.

At higher temperatures, the disordered fluid undergoes a continuous order–disorder transition, up to the temperatures corresponding to the order–disorder transition in close-packed systems. Upon a further lowering of $u_{BB}$ below about −0.116, the phase behavior changes, and the first order transition between the gas-like phase, and the condensed phase was found to lead directly to the ordered zigzag structure only at temperatures below the critical end point, which was the onset of the continuous order–disorder transition.

**Figure 13:**
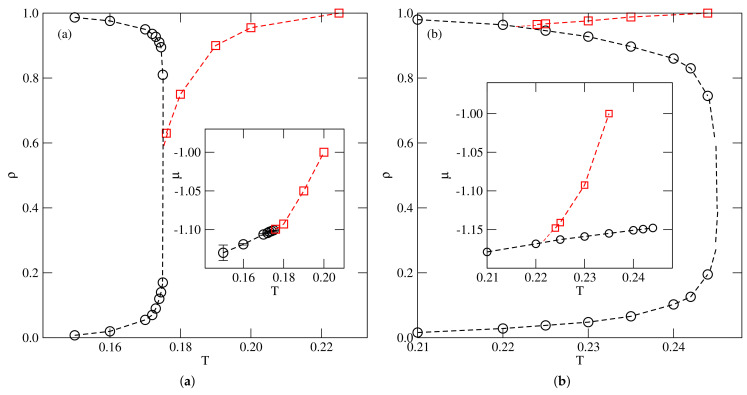
The estimated phase diagrams for the systems with $u_{AB}=0$ and $u_{bB}$ equal to −0.10 (**a**) and −0.2 (**b**). The main panels and insets show the T−ρ and the T−μ projections, respectively. The circles and squares mark coexistence points of first−order and continuous transitions, respectively.

**Figure 14:**
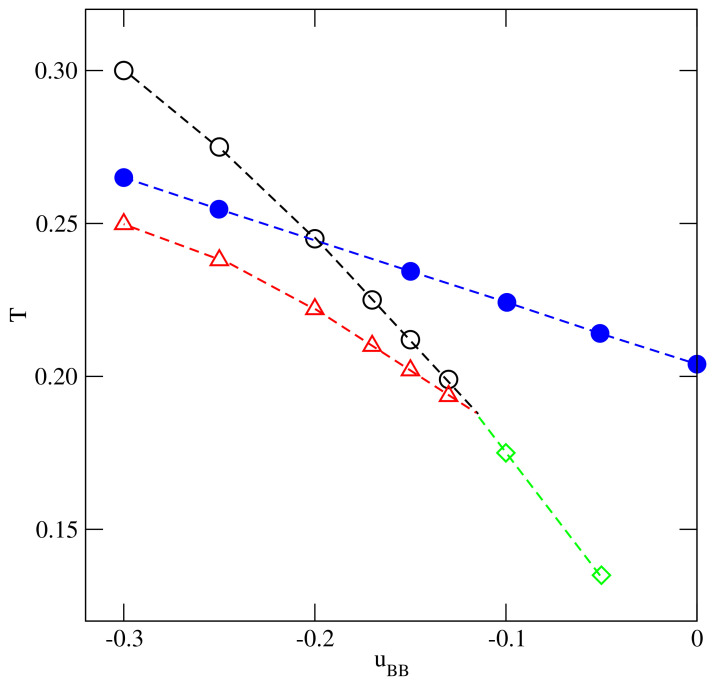
The changes of the critical temperature, $T_{c}\left(u_{BB}\right)$, (circles), the tricritical point temperatures, $T_{trc}\left(u_{BB}\right)$ (diamonds), the critical end point temperatures, $T_{cep}\left(u_{BB}\right)$ (squares), and the orientational order−disorder transition temperature in close-packed systems, $T_{o}\left(u_{BB}\right)$ (filled circles), for the systems with $u_{AB}=0$.

Above the critical end point temperature, the gas-like phase condenses into the disordered lamellar liquid, and the transition terminates in the critical point. The critical end point temperature and the critical temperature were found to increase when $u_{BB}$ decreased. Figure 13 presents the examples of phase diagrams obtained for $u_{BB}=-0.1$ (part a) and −0.2 (part b), which demonstrate the changes of the topology with $u_{BB}$, while Figure 14 shows the estimated changes of $T_{c}$, $T_{cep}$, $T_{trc}$ and $T_{o}$ with $u_{BB}$.

It is well seen that $T_{c}$ and $T_{cep}$ meet $T_{trc}$ at $u_{BB}\approx -0.116$. Thus, for any $u_{BB}$ lower than about −0.116, the phase diagram topology is expected to remain unchanged. When $u_{BB}$ decreases, the critical end point temperature gradually approaches the temperature at which the close-packed systems undergo the order–disorder transition. We limited the calculations to $u_{BB}$ down to −0.3, since the estimation of the critical end point temperatures for lower values of $u_{BB}$ was quite difficult. Already for $u_{BB}=-0.3$, $T_{cep}\left(-0.3\right)$ is quite close to $T_{o}\left(-0.3\right)$, and the density at the critical end point is quite high and equal to about 0.98.

In the series with $u_{BB}=0$, the nature of the order–disorder transition in dense systems is different for $u_{AB}$ lower and higher than −0.5. Namely, when $u_{AB}>-0.5$, this transition belongs to the universality class of the three-states Potts model, while, for $u_{AB}<-0.5$, it belongs to the universality class of the four-state Potts model [20]. In the particular case of $u_{AB}=-0.5$, the orientational order–disorder transition does not occur at all. In the close-packed systems, the temperature of the order–disorder transition decreases from 0.204, when $u_{AB}=0$ to zero, when $u_{AB}=-0.5$. Then, for $u_{AB}<-0.5$, the transition temperature increases from zero up to about 0.14, when $u_{AB}$ decreases from −0.5 to −1.0.

**Figure 15:**
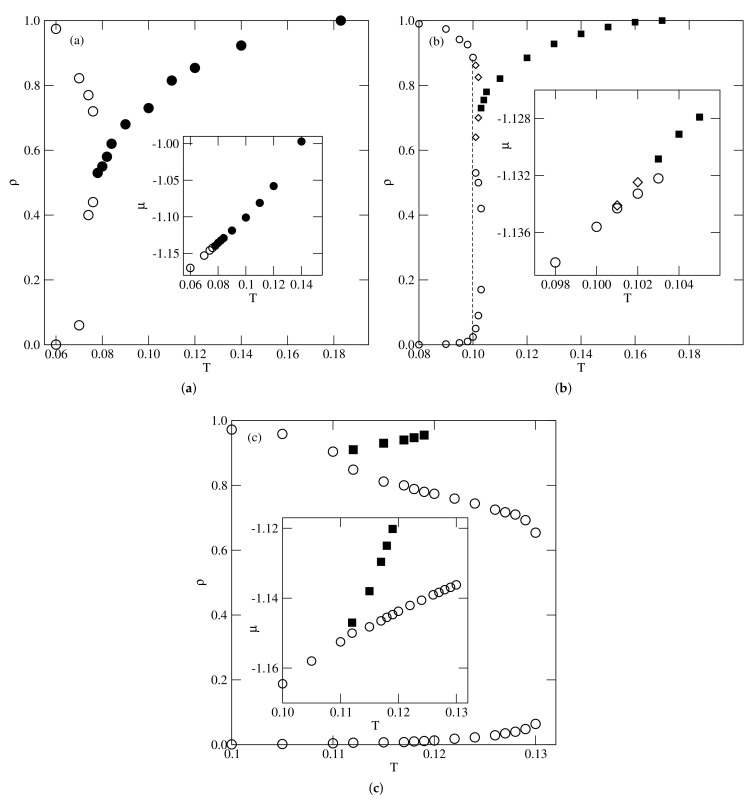
The estimated phase diagrams for the systems with $u_{BB}=0$ and different values of $u_{AB}$, equal to −0.05 (**a**), −0.1 (**b**) and −0.15 (**c**). The main figures and insets show the T−ρ and the T−μ projections, respectively. The vertical line in part b marks the location of the triple point. Open and filled symbols correspond to the first−order and continuous transitions, respectively. In part b, the low and high density transitions are marked by circles and diamonds, respectively.

Here, we studied only the systems with $u_{AB}$ between −0.05 and −0.25, and Figure 15 presents three phase diagrams obtained for $u_{AB}=-0.05$, −0.1, and −0.15. In the case of $u_{AB}=-0.05$, the phase behavior is qualitatively the same as in the already discussed systems with $u_{AB}=u_{BB}=-0.03$ and −0.05, as well as in the systems with $u_{AB}=0$ and $u_{BB}=-0.05$ and −0.1. Thus, the fluid phase condenses directly into the ordered zigzag structure at any temperature, between zero and the temperature at which the close-packed system undergoes the order–disorder transition.

In the case of $u_{AB}=-0.1$, the dilute gas-like phase condenses into the ordered zigzag phase, only at temperatures up to the triple point temperature, $T_{tr}\left(-0.1\right)\approx 0.1$, At slightly higher temperatures, up to about 0.103, the gas condenses into the disordered liquid phase. The transition terminates at the usual critical point. However, at temperatures between about 0.1 and 0.102, the disordered liquid undergoes the first-order transition to the ordered zigzag phase. At ≈0.102, the tricritical point appears, and, at still higher temperatures, the disordered fluid undergoes a continuous transition to the zigzag phase.

The system with $u_{AB}=-0.15$ shows qualitatively different phase behavior, and the onset of a continuous order–disorder transition is located in the critical end point. Here, again, at the temperatures above the critical end point and up to the critical point, the gas-like phase condenses into the disordered liquid. Thus, the behavior is the same as in the already discussed systems with $u^{*}<-0.076$ and with $u_{AB}=0$ and $u_{BB}<-0.116$.

**Figure 16:**
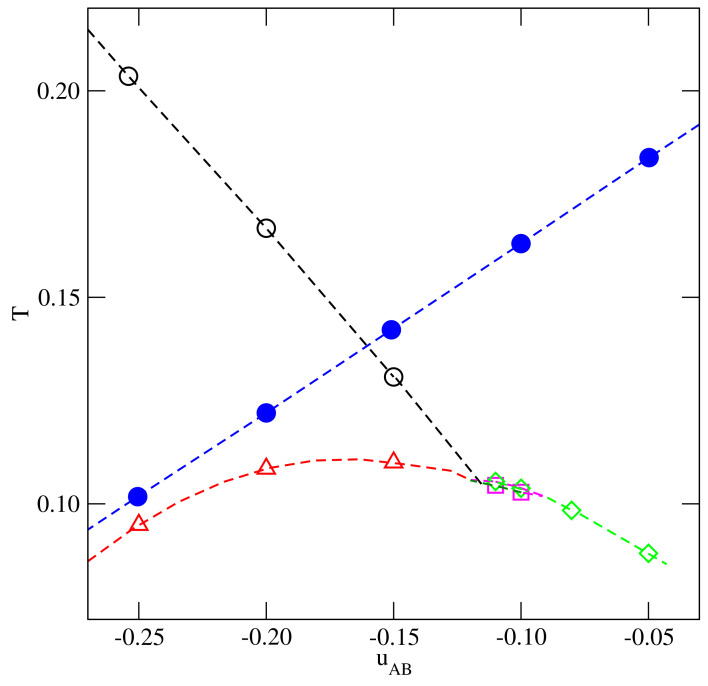
The changes of the $T_{c}\left(u_{AB}\right)$, (circles), $T_{trc}\left(u_{AB}\right)$ (triangles), $T_{cep}\left(u_{AB}\right)$ (diamonds), $T_{tr}\left(u_{AB}\right)$ (squares), and $T_{o}\left(u_{AB}\right)$ (filled circles) estimated for the series with $u_{BB}=0$.

From the calculations carried out for several systems, we can estimate the changes of $T_{c}$, $T_{cep}$, $T_{trc}$ and $T_{tr}$ with $u_{AB}$, shown in Figure 16. Similarly to previously discussed systems, the tricritical point and the critical point temperatures increase when the AB attraction becomes stronger, and these two regimes meet when $u_{AB}\approx -0.116$. However, the critical end point temperature exhibits non-monotonous changes with $u_{AB}$. The temperature of the order–disorder transition in a close-packed system. $T_{o}\left(u_{AB}\right)$, decreases when the AB attraction becomes stronger, and $T_{cep}\left(u_{AB}\right)$ is bound to be lower than $T_{o}\left(u_{AB}\right)$.

## 4. Summary

We studied the phase behavior of two-dimensional systems of Janus-like particles on a triangular lattice. Here, we assumed that all, AA, AB, and BB, interactions are attractive. The AA interaction energy was fixed, while the AB and/or BB interaction energies were varied, and assumed to be less attractive than the AA interaction. We assumed that the particles can take on only six different orientations and that the interaction energy between a pair of nearest neighbors depends on their mutual orientations. Using the grand canonical Monte Carlo simulation method, we considered three series of systems with $u_{AB}=u_{BB}$, $u_{AB}=0$ and with $u_{BB}=0$.

We demonstrated that the phase behavior of all systems strongly depends on the stability of the high density zigzag (Z) phase. The stability of the Z phase is determined by the anisotropy of interactions and increases when the AB and/or BB attractions become weaker. As a consequence, in the systems with sufficiently strong anisotropy of interactions, the liquid phase does not appear, and the dilute fluid condenses directly into the zigzag ordered phase. The transition terminates in the tricritical point. At temperatures above the tricritical point, the disordered fluid undergoes a continuous transition into the zigzag phase.

When the AB and/or BB attraction increases, the stability of the zigzag phase becomes weaker, and its formation is possible only at sufficiently high densities and at sufficiently low temperatures. This means that the dilute phase condenses into the zigzag phase only at the temperatures lower than the critical end point temperature. At temperatures above the critical end point, the dilute phase condenses into the disordered liquid-like phase, and the transition terminates in the usual critical point.

The above scenario was found in all three considered series of systems. Whenever the critical point appears, the critical temperature increases when the attraction between AB and/or BB halves becomes stronger. In the particular series with $u_{AB}=u_{BB}=u^{*}$, the critical temperature went up to the value corresponding to the critical point of the uniform system when $u^{*}=-1.0$. In the series with $u_{AB}=0$ and with $u_{BB}=0$, the critical temperatures reached lower values when $u_{BB}$ or $u_{AB}$ went to −1.0.

In the case of the series with $u_{AB}=0$, the phase diagram topology remained the same for any $u_{BB}$ lower than about −0.116. Thus, the onset of the continuous order–disorder transition in the dense fluid meeting the bulk coexistence in the critical end point, $T_{cep}\left(u_{BB}\right)$, and $T_{cep}\left(u_{BB}\right)$ gradually increased when $u_{BB}$ is lowered. On the other hand, the series with $u_{BB}=0$ is expected to show different behavior, when $u_{AB}$ decreases. In this paper, we discussed only the systems with $u_{AB}\geq -0.25$.

In this series, the critical end point temperature, $T_{cep}\left(u_{AB}\right)$, is bound to go to zero for $u_{AB}=-0.5$, since this particular system does not undergo any orientational order–disorder transition [20]. However, a further decrease of $u_{AB}$ below −0.5 means that the order–disorder transition reappears; however, now, this transition belongs to the universality class of the four-state Potts model. Therefore, it is expected that the continuous order–disorder transition should occur at sufficiently high densities and at sufficiently low temperatures. The onset of this transition is also expected to be located at the critical end point, $T_{cep}\left(u_{AB}\right)$.

Here, we recall the results obtained for symmetric mixtures [27,28], which show qualitatively the same changes in the phase diagram topology when the tendency toward demixing becomes weaker. In that case, the demixed fluid is an ordered state, and by lowering its stability, the same sequence of phase diagram topologies appears.

## Figures and Tables

**Table 1:** Possible different values of the interaction energy between a pair of neighboring particles and the pairs of orientations of a given energy for the separation vector vecr=(1,0).

$u_{1}$ =	$u_{AA}$	(0,3)
$u_{2}$ =	$u_{AB}$	(0,0), (1,5), (2,4), (3,3), (4,2), (5,1)
$u_{3}$ =	$u_{BB}$	(3,0)
$u_{4}$ =	$0.75u_{AA}+0.25u_{AB}$	(0,2), (0,4), (1,3), (5,3)
$u_{5}$ =	$0.75u_{AA}+0.25u_{BB}$	(1,2), (5,4)
$u_{6}$ =	$0.75u_{BB}+0.25u_{AB}$	(2,0), (3,1), (3,5), (4,0)
$u_{7}$ =	$0.75u_{BB}+0.25u_{AA}$	(2,1), (4,5)
$u_{8}$ =	$0.75u_{AB}+0.25u_{AA}$	(0,1), (0,5), (2,3), (4,3)
$u_{9}$ =	$0.75u_{AB}+0.25u_{BB}$	(3,2), (5,0), (3,4), (1,0)
$u_{10}$ =	$0.5u_{AB}+0.25\left(u_{AA}+u_{BB}\right)$	(1,1), (2,2), (4,4), (5,5)
$u_{11}$ =	$0.5u_{AA}+0.5u_{AB}$	(1,4), (5,2)
$u_{12}$ =	$0.5u_{BB}+0.5u_{AB}$	(2,5), (4,1)

## Data Availability

Not applicable.
